# Safety and Utility of Smartphone‐Based Heart Monitors in Pediatric Patients With Cardiovascular Implantable Electronic Devices

**DOI:** 10.1111/jce.70444

**Published:** 2026-07-19

**Authors:** Chun‐Lok Ho, Sit‐Yee Kwok, Sabrina S. L. Tsao, Yiu‐Fai Cheung

**Affiliations:** ^1^ Department of Pediatrics and Adolescent Medicine Princess Margaret Hospital Hong Kong China; ^2^ Department of Pediatrics and Adolescent Medicine Hong Kong Children's Hospital Hong Kong China; ^3^ Department of Pediatrics and Adolescent Medicine, School of Clinical Medicine, LKS Faculty of Medicine The University of Hong Kong Hong Kong China

**Keywords:** AliveCor, Apple Watch electrogram, electromagnetic interference, epicardial device, non‐capture, personal ECG device

## Abstract

**Background:**

Portable heart monitors enable on‐demand electrocardiogram (ECG) recordings and enhance symptom‐rhythm correlation. However, concerns have been raised regarding potential electromagnetic interference (EMI) from these monitors that may affect cardiovascular implantable electronic devices (CIEDs). The safety and clinical benefits of portable heart monitors in children and adolescents with implanted CIEDs remain to be established.

**Objectives:**

The primary objective was to determine whether KardiaMobile 6L and Apple Watch induce EMI, impacting the function of CIEDs in pediatric patients. Secondary aims included the assessment of the accuracy of heart rate measurement and their ability to detect non‐capture events.

**Methods:**

In this cross‐sectional study, patients aged 5 years or older with implanted CIEDs were recruited from a tertiary pediatric cardiac center. Each subject had four 30 s ECGs acquired using KardiaMobile 6L and Apple Watch. Subjects were monitored for cardiac symptoms during ECG acquisition, and CIEDs were assessed for EMI. The collected ECGs were analyzed for interpretability, accuracy of heart rate measurement, and detection of non‐capture events.

**Results:**

Thirty‐two patients (median age 14.0 years, IQR 9.9–16.6) were recruited. No adverse clinical events were identified. No EMI was detected during ECG acquisition, regardless of patient and CIED characteristics. Ninety‐eight percent of acquired ECGs were interpretable. Both devices demonstrated excellent accuracy of heart rate measurement (*ρ* = 0.99) and a sensitivity of 100% for identifying ventricular non‐capture events.

**Conclusions:**

KardiaMobile 6L and Apple Watch did not induce EMI in children and adolescents with CIEDs, enabling reliable heart rate measurement and accurate identification of ventricular non‐captures on these smartphone‐based portable heart monitors.

## Graphical abstract

Smartphone heart monitors in pediatric CIEDs: A pilot study. Smartphone heart monitors did not induce EMI in children with CIEDs, enabling reliable heart rate measurement and accurate identification of ventricular non‐captures.

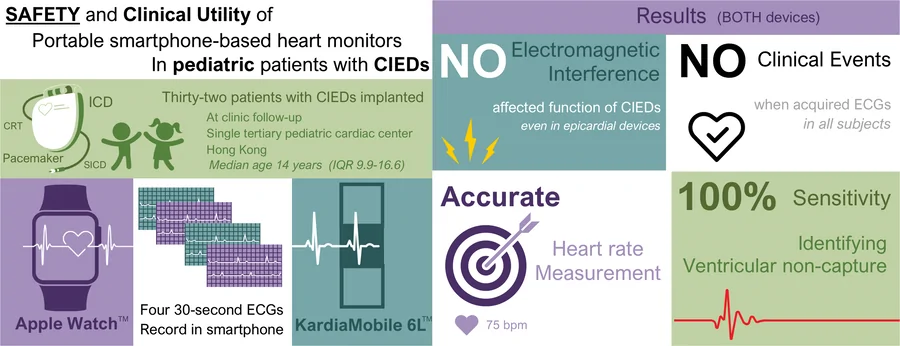

**Abbreviations**
CIED(s): cardiovascular implantable electronic device(s)
CRT: cardiac resynchronization therapy
ECG: electrocardiogram
EGM: electrogram
EMI: electromagnetic interference
ICD: implantable cardioverter defibrillator
IQR: interquartile range
RM: remote monitoring
SICD: subcutaneous implantable cardioverter defibrillator

## 1 Introduction

Cardiovascular implantable electronic devices (CIEDs) in children differ from adults due to the heterogeneity in the underlying cardiac anomalies and limitations of vascular access. Epicardial lead implantation is frequently required [1]. Epicardial leads are associated with lower lead impedance, earlier lead failure, and hence depletion of battery life because of the increased pacing threshold [2]. Young children have difficulty communicating symptoms if device issues or arrhythmias exist. Although remote home transmissions for CIEDs enable frequent assessment of lead function, remote monitoring (RM) and in‑office interrogation cannot detect intermittent, infrequent episodes of ventricular non‑capture events that may be clinically important [3]. Therefore, a convenient on‑demand heart rhythm monitor is essential to improve the detection of arrhythmias and device malfunctions, particularly when patients experience symptoms.

Recently, portable or wearable digital devices have been widely used to provide on‐demand electrocardiogram (ECG) recordings, with demonstrable symptom‐rhythm correlation in children [4, 5]. Webster et al. demonstrated that digital devices can cause interference to the CIED interrogation, with noise detected in the ECG channel in programmers [6]. Hence, there have been ongoing concerns regarding the use of these devices with CIEDs for the potential electromagnetic interference (EMI) due to the emitted electromagnetic radiation. EMI can manifest as noise in CIEDs and compromise their function [6, 7, 8].

Currently, there are two commonly used portable smartphone‐based heart monitors. One is KardiaMobile (AliveCor, Mountain View, CA), which has been shown to produce no EMI in adult studies [9]. ECGs generated are widely utilized in various clinical settings [10, 11]. The other device is Apple Watch (Apple Inc, Cupertino, CA), which can generate ECGs in children with or without congenital heart disease [12]. As the Apple Watch contains a magnet, keeping the watch at least 15 cm away from medical devices is recommended to mitigate potential interference [13].

To date, no studies have investigated the use of portable smartphone‐based heart monitors in pediatric patients with CIEDs. The primary aim of this study was to determine whether KardiaMobile 6L and Apple Watch induce EMI that may have an impact on the function of CIEDs in pediatric patients. The secondary aims were to assess the accuracy of heart rate measurement and the ability of these portable heart monitors to detect non‐capture events.

## 2 Methodology

### 2.1 Subjects

This was a cross‐sectional study of patients aged 5 years or older and followed up in the cardiology clinic at a tertiary pediatric cardiac center. Patients with CIEDs implanted were recruited during clinic follow‐up. Exclusion criteria included the following: (i) pacemaker dependence, (ii) presence of symptoms including chest pain, palpitations, or dizziness at the time of recruitment, and (iii) device malfunction during the initial device interrogation. Patients aged 18 years or older who were following up in the clinic and awaiting transfer to adult cardiology were not excluded from this study to maximize sample size. All adult patients and parents of pediatric subjects gave written informed consent. The study was approved by the Central Institutional Review Board of the Hospital Authority, Hong Kong.

The following data were collected for each of the subjects: (i) patient demographics, (ii) clinical data: indication of device implantation, primary arrhythmia diagnosis, associated structural cardiac abnormalities, and (iii) details of the CIED device: type and brand, time from installation, device location, lead type, and pacing mode. The body weight and height were measured. A 12‐lead ECG was obtained before device interrogation.

### 2.2 CIED Monitoring During the Use of Portable Heart Monitors

All of the subjects first underwent the usual device interrogation to ensure normal operation of the implanted devices. Defibrillation and overdrive pacing functions of CIEDs were then temporarily turned off during the study to prevent possible false triggers of therapy by EMI. During ECG acquisition by KardiaMobile 6L and Apple Watch, the following events were monitored: (i) new onset of cardiac symptoms including chest pain, palpitations, or dizziness, and (ii) presence of EMI by real‐time monitoring of near‐field and far‐field intracardiac electrogram (EGM) and in the printed strips. The EGMs were assessed for artifacts, noise, oversensing or undersensing, and signal loss due to EMI. Medical staff accompanied each subject throughout the study period.

### 2.3 Acquisition of ECG Using Apple Watch and KardiaMobile 6L

For ECG acquisition using the Apple Watch Series 8, the subject was seated with the watch on the left wrist at least 20 cm from the trunk of the body and the CIED. The subject was then instructed to place his or her right index finger onto the crown of the watch to obtain a 30 s recording of a lead I ECG. The Apple Watch was paired to an iPhone SE3 (Apple Inc, Cupertino, CA). The ECG tracings, which had no identifiable personal information, were stored for further evaluation. A second ECG tracing was acquired during pacing threshold tests to determine whether the device can assess non‐capture. The pacing output of the pacing leads (either atrial or ventricular lead) was gradually lowered until non‐capture.

To acquire a 6‐channel ECG (lead I, II, III, aVF, aVR, aVL) using KardiaMobile 6L, the subject was instructed to hold the device with both thumbs touching the top electrodes and place it on the bare skin of the left knee or inside the left ankle, whichever was most comfortable, in a seating position. A second ECG tracing was again acquired during the pacing threshold tests.

The acquisition of ECG would be terminated immediately in the event of an EMI or when the subject reported clinical symptoms. After completion of ECG acquisition, the CIED was interrogated again for any alarm triggers, device warnings, and changes in program settings, the latter including mode switching, safety pacing, noise‐reversion mode, asynchronous pacing, inhibition pacing, and magnet mode.

### 2.4 Review of the ECG and EGM Tracings

The EGMs from the device interrogation, ECG tracings from KardiaMobile 6L and Apple Watch were reviewed for the followings: (i) accuracy of heart rate measurements: the average heart rate (ventricular rate) automatically calculated by the representative mobile applications were compared with the average heart rate directly measured from simultaneously recorded EGM strips, (ii) interpretability: two investigators (C.L.‐H. and S.S.L.T.) reviewed the KardiaMobile 6L and Apple Watch ECG tracings to determine their interpretability, and (iii) detection of non‐capture: an electrophysiology specialist (S.S.L.T) blinded to the subject and device information reviewed the ECG tracings obtained with KardiaMobile 6L and Apple Watch during pacing threshold tests. During the analysis, the reviewer was not informed whether the ECG tracings represented atrial or ventricular pacing threshold testing. Non‐capture events were identified by observing ECG changes, including prolonged pauses, escape beats, heart rate variations, and alternations in P or QRS morphologies.

### 2.5 Statistical Analysis

Data are reported as median, range, and interquartile range (IQR). The agreement of heart rate measurements was evaluated using Bland−Altman plots and Pearson's correlation analysis. A *p* value less than 0.05 was considered statistically significant. Statistical analyses were performed using SPSS version 29. In the absence of observed events, the maximum risk can be determined with a 5% error by solving 1−(0.05)^1/*n*^ [n = number of subjects]. By Hanley's simple formula, when the number of subjects is more than 30, the maximum risk can be approximated by 3/*n* [14].

## 3 Results

### 3.1 Patient Demographics

A total of 32 subjects (21 males and 11 females) met study criteria and were recruited. None of the patients had cardiac symptoms during the device check. No device malfunction was observed during the initial CIED interrogation. Their median age was 14.0 years (IQR, 9.9−16.6), ranging from 5.2 to 26.7 years. The median weight of the study population was 44 kg (IQR, 30−57 kg), while the median height was 153 cm (IQR, 138−167 cm). The median body mass index was 17.9 kg/m^2^, ranging from 12.8 to 30.2 kg/m^2^ (IQR, 15.8−21.0 kg/m^2^). Sixteen (50%) of the recruited patients had underlying structural heart disease which included ventricular septal defect (*n* = 4), atrioventricular septal defect (*n* = 2), tetralogy of Fallot (*n* = 2), dilated cardiomyopathy (*n* = 2), hypertrophic cardiomyopathy (*n* = 2), double‐outlet right ventricle with pulmonary stenosis (*n* = 1), Ebstein anomaly (*n* = 1), pulmonary atresia with ventricular septal defect (*n* = 1), and complete transposition of great arteries (*n* = 1). Congenital and acquired heart block were the most common underlying rhythm disorders (Table 1).

**Table 1:** Types of CIEDs and their configurations and underlying rhythm disorders.

Rhythm disorders	Type of CIEDs and configuration						
	Single chamber		Dual Chamber		CRT		SICD
	AAI (R)	VVI (R)	DDD (R)	ICD	CRT‐P	CRT‐D	
Aborted ventricular fibrillation arrest						1	1
Acquired complete heart block		1	4		1		
Catecholaminergic polymorphic ventricular tachycardia (CPVT)				3			
Congenital heart block		3	4	1			
Left bundle branch block with ventricular dyssynchrony					1		
Long QT syndrome		1		4			
Primary prevention ICD for hypertrophic cardiomyopathy							1
Sinus node dysfunction	1	2 (leadless: 1)	3				

### 3.2 Device Characteristics

Nineteen subjects (60%) had pacemakers, 8 (25%) had implantable cardioverter defibrillators (ICD), 3 subjects (9.4%) had cardiac resynchronization therapy (CRT) devices, and 2 (6.3%) had subcutaneous implantable cardioverter defibrillators (SICD). Sixteen (50%) patients had transvenous leads, while 13 (40%) had epicardial leads. One subject had a leadless pacemaker implanted. Twenty‐six generator models from three different manufacturers were included. Devices were implanted in the left pectoral area in 16 subjects (50%), the abdomen in 13 (40.6%), the right pectoral area in 1 (3.1%), and the left axillary region in 1 (3.1%). Nineteen (60%) subjects were in a paced rhythm at the time of study. Notably, the median sensitivity settings of atrial and ventricular leads were 0.30 mV (IQR 0.30–0.45) and 0.90 mV (IQR 0.68–2.00), respectively. All ICD ventricular leads were programmed at bipolar sensing. The time from CIED implantation ranged from 1 to 129 months, with a median duration of 34 months.

### 3.3 Clinical Events

No adverse clinical events, including chest pain, palpitations, or dizziness, were reported by the subjects during the KardiaMobile 6L ECG and Apple Watch ECG recordings.

### 3.4 Safety Analysis

No EMI was observed. The review of near‐field and far‐field intracardiac EGM revealed no evidence of noise, oversensing, undersensing, or signal loss. Furthermore, no warnings, alarms, or changes in device settings or modes occurred during the recording of ECGs. Applying Hanley's simple formula, the 95% confidence interval for this zero‐event observation ranged from 0% to 9.4%.

### 3.5 Accuracy of Heart Rate Measurement

There were no significant systematic differences between the measurements demonstrated in the Bland−Altman plots for KardiaMobile 6L (Figure 1) and Apple Watch (Figure 2). The Pearson's coefficient (*ρ*) was 0.995 for KardiaMobile 6L and 0.993 for Apple Watch, showing a strong correlation between the heart rate measured by either of the devices and their simultaneous EGM.

**Figure 1:**
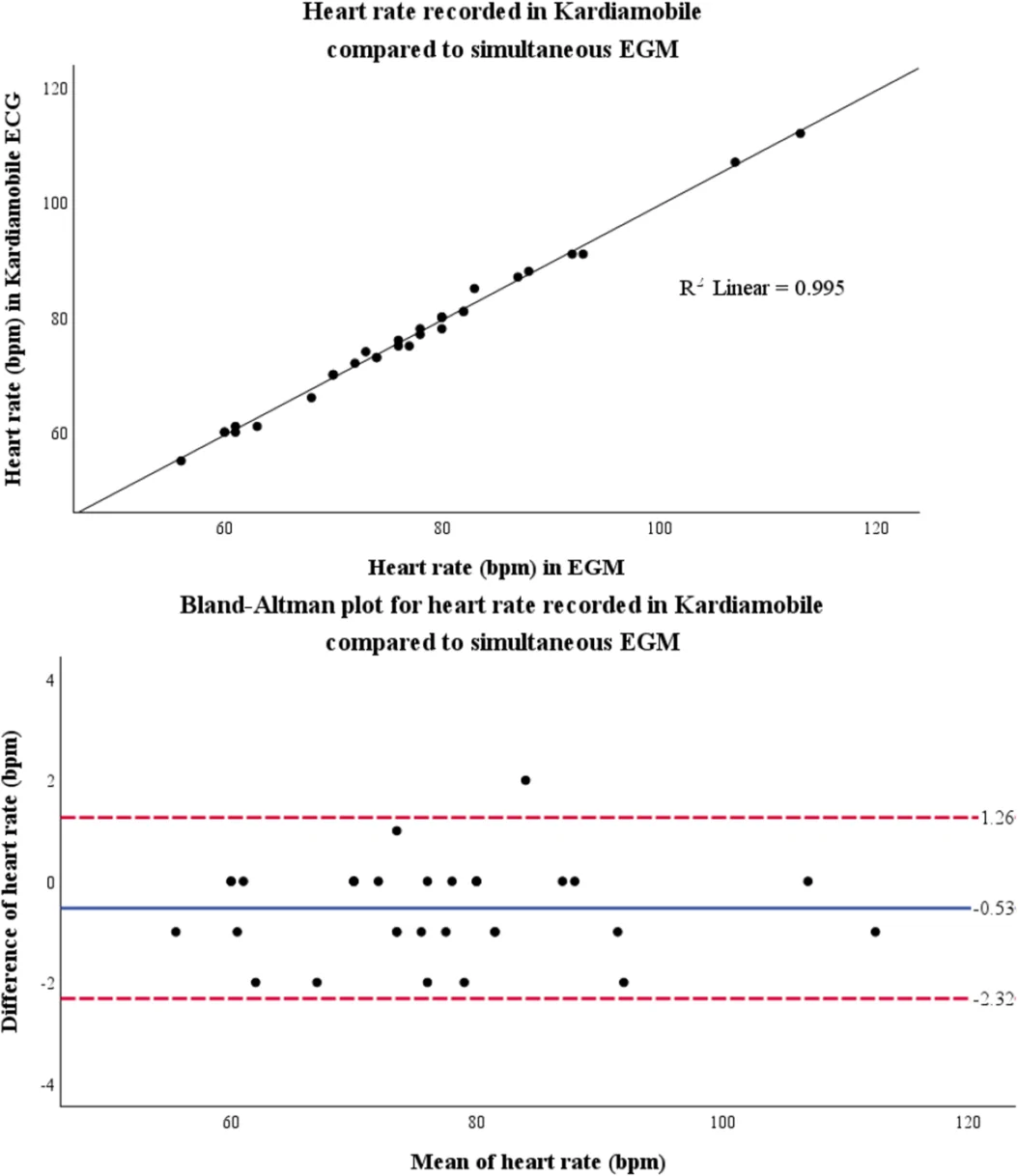
Scattered plot and Bland−Altman plot of heart rate measurement in KardiaMobile 6L compared with simultaneous EGM.

**Figure 2:**
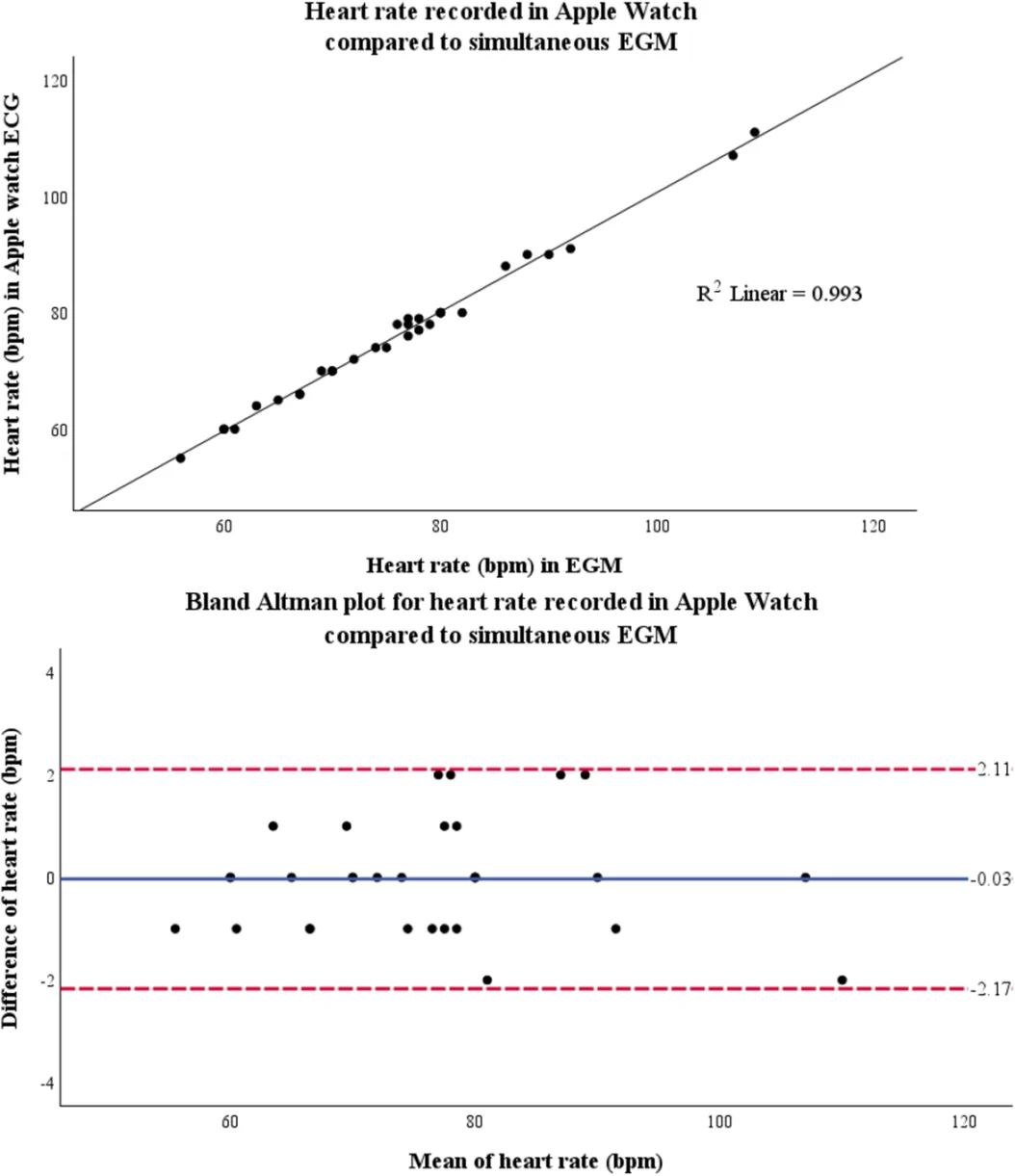
Scattered plot and Bland−Altman plot of heart rate measurement in Apple Watch compared with simultaneous EGM.

### 3.6 Interpretability

All ECGs recorded with Apple Watch were found to be interpretable. All ECGs but one recorded with KardiaMobile 6L were interpretable. The uninterpretable ECG was related to motion artifacts.

### 3.7 Identification of Non‐Captures

Twenty subjects recorded the second ECGs during ventricular threshold tests, while eight subjects recorded the second ECGs during atrial threshold tests (Table 2). Pacing threshold tests were not indicated in the two SICD subjects. One subject with an ICD could not acquire ECGs using both the Apple Watch and KardiaMobile 6L during the pacing threshold test and was excluded from the analysis*. Another subject failed to obtain an ECG record with the Apple Watch during the ventricular threshold test*, and therefore, only the ECG recorded from the KardiaMobile 6L was analyzed. One subject with a pacemaker was excluded from the analysis because the programmed safety pacing mode during the ventricular threshold test obscured non‐capture events in the EGM. *(The two subjects couldn't hold the KardiaMobile or Apple Watch securely to complete the recording during the ventricular threshold test; they lost patience and declined further ECG recordings).

**Table 2:** ECG recorded during pacing threshold test.

	KardiaMobile 6 L	Apple Watch
Total number of subjects	32	32
Include		
Successful ECG record in ventricular threshold test	20	19
Successful ECG record in atrial threshold test	8	8
Exclude		
Subjects with SICD	2	2
No tracing obtained in pacing threshold test	1	1
Device was in ventricular safety pacing during test	1	1
Subject did not acquire ECG in ventricular threshold test (due to technical issues)	0	1

Among the ECGs obtained during the ventricular threshold tests, non‐capture events were correctly identified in all ECGs recorded by both devices, demonstrating a sensitivity of 100% for ventricular non‐captures (Figure 3). By contrast, atrial non‐capture events were correctly identified in only six out of the eight ECGs recorded with the KardiaMobile 6L and seven out of the eight ECGs recorded with the Apple Watch. The sensitivity for atrial non‐captures was 75% for KardiaMobile 6L and 88% for Apple Watch, respectively. The reviewer could not identify an atrial non‐capture event in one ECG captured by KardiaMobile 6L because the long pause extended across the change of pages in PDF (Figure 4). The other two cases were not identified due to motion artifacts.

**Figure 3:**
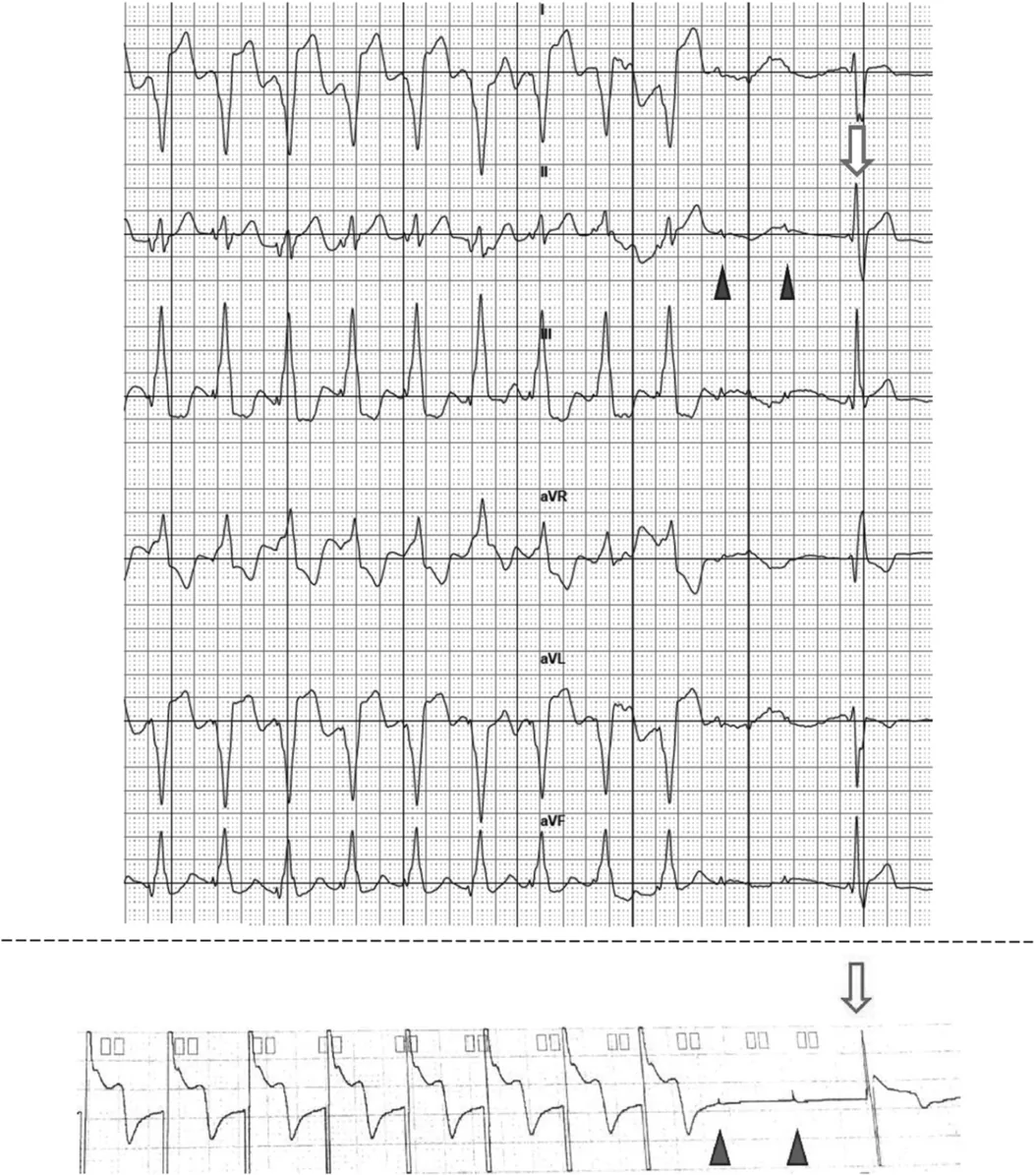
Detection of ventricular non‐capture with KardiaMobile 6L. Illustration of ECG (upper panel) and EGM (lower panel) tracings during ventricular pacing threshold test in a patient with complete heart block and unipolar epicardial pacemaker, showing non‐captures (triangles) and junctional escape beat (hollow arrow).

Figure 4Missed atrial pacing event in KardiaMobile 6L ECG. This highlighted excerpt demonstrates a missed atrial pacing event with a marked RR interval prolongation from 920 to 1777 ms, spanning two pages during the initial review in the PDF.

**Figure:**
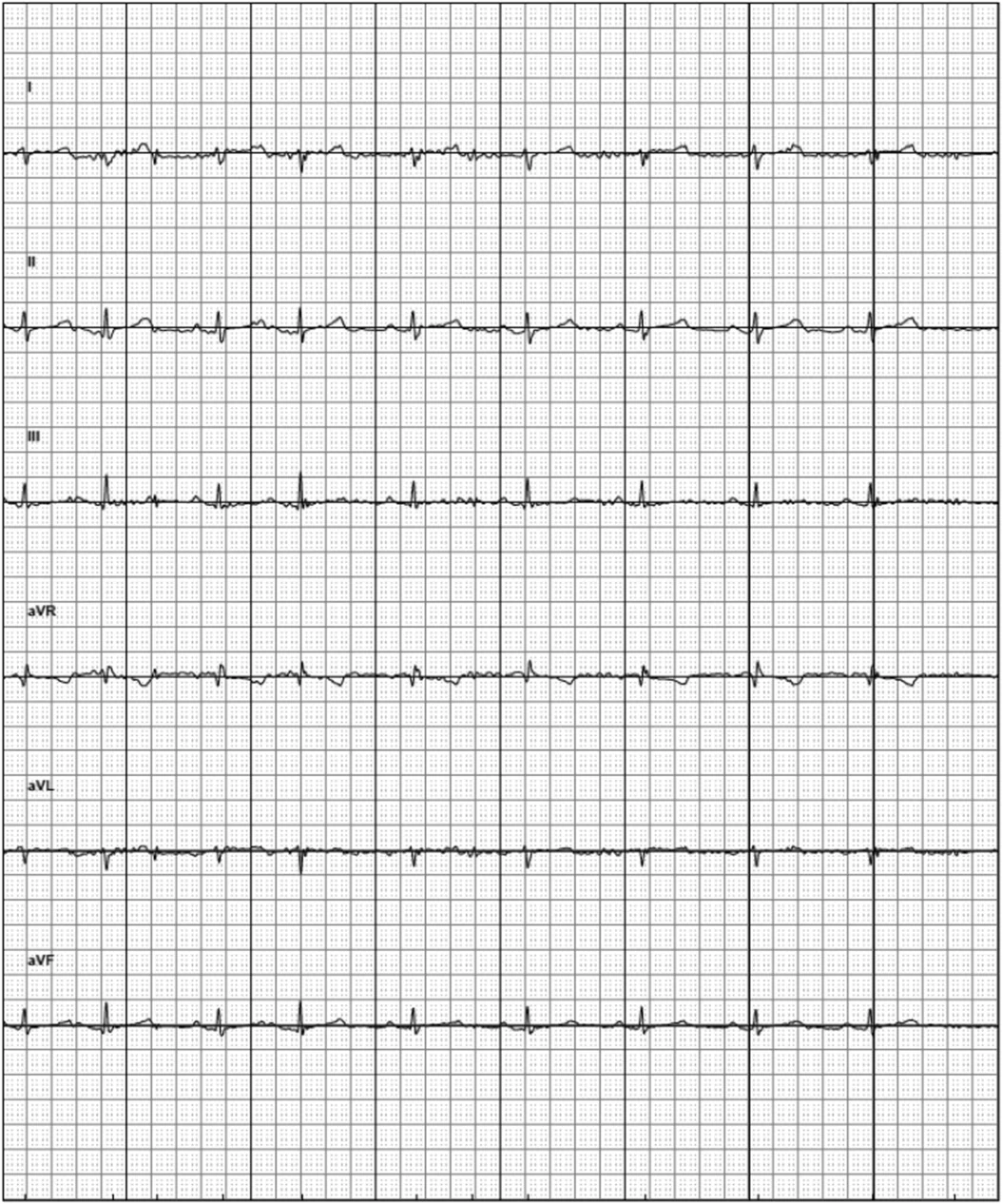

**Figure:**
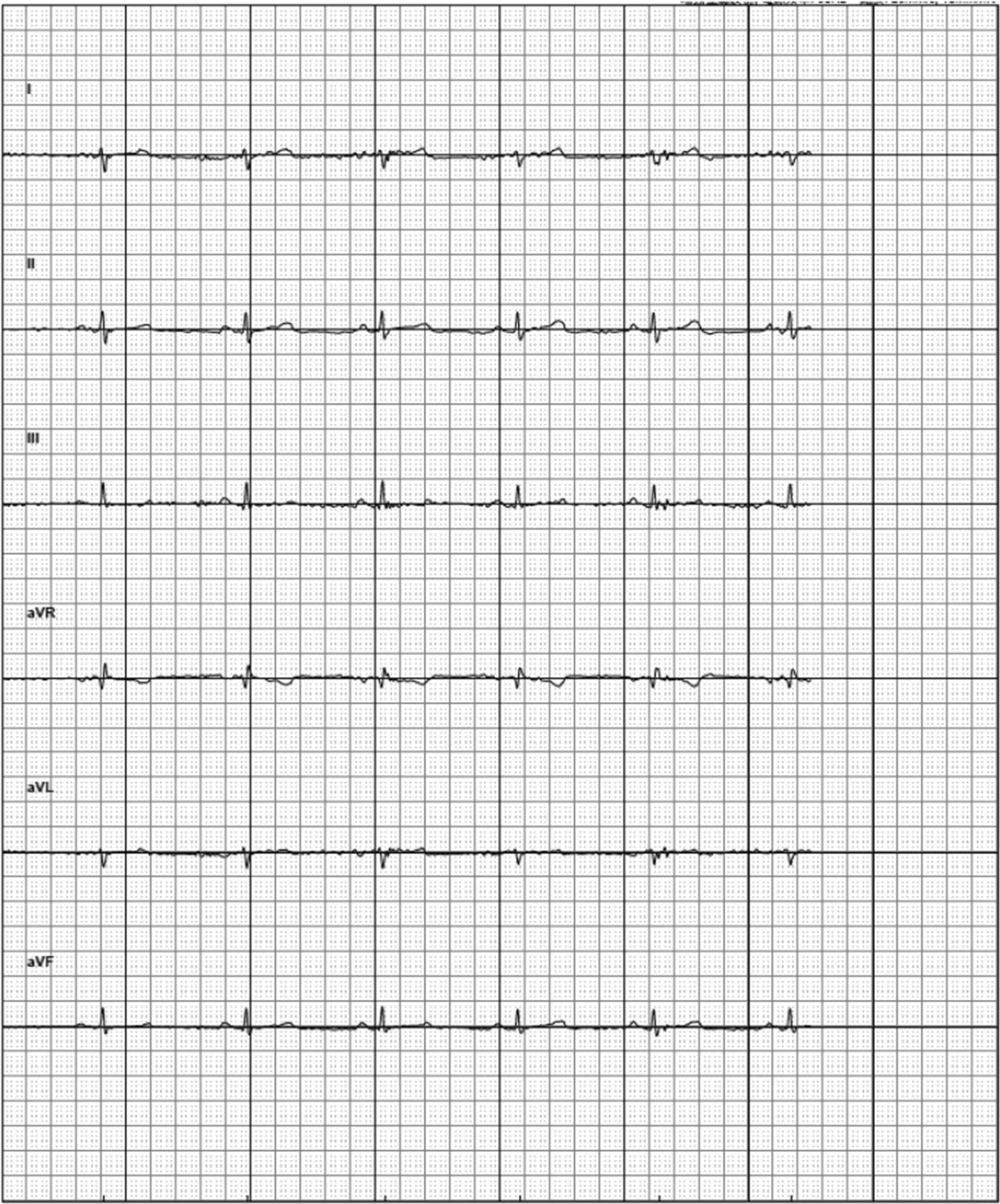

## 4 Discussion

### 4.1 Major Findings in Our Study

To the best of our knowledge, this study represents the first attempt to investigate the safety and potential clinical applications of portable or wearable smartphone‐based heart monitors in the pediatric population with CIEDs. No EMI was detected even in children with epicardial pacing systems, in which the abdominal generators were close to these heart monitors. These outcomes remained consistent across subjects with varying body builds, with or without congenital heart diseases, and different types of CIEDs from different manufacturers. While the lack of observed EMI suggests that routine interference is unlikely, the study was not powered to detect rare adverse events. On the other hand, KardiaMobile 6L and Apple Watch demonstrated excellent accuracy in measuring heart rate. They produced ECGs that closely resembled those from standard 12‐lead ECG machines, enabling the identification of non‐capture events and rhythm changes.

### 4.2 Evidence From Previous Studies

Modern CIEDs and wearables have significantly improved to mitigate the functional impact of EMI. Modern CIEDs utilize titanium housing for physical shielding, feedthrough filtered capacitors, and bipolar leads to minimize antenna effects, alongside advanced digital signal processing to filter out noise [15]. Despite these advancements, in vivo studies showed that smartphones and wearables with high‐density magnets can induce magnet mode in devices equipped with magnetically sensitive switches [16, 17]. Furthermore, wearables/personal well‐being devices utilizing bioimpedance to transmit electrical currents through the body have been shown to cause oversensing, which may lead to pacing inhibition in patients with CIEDs [18].

Clinical studies have focused on the determination of the safety of portable or wearable smartphone‐based heart monitors in adults with CIEDs. Abudan et al. reported the absence of clinical events or evidence of EMI in 251 adult patients when using AliveCor Kardia device to record ECGs [9]. Similarly, Lacour et al. found no evidence of EMI in 148 adult CIED patients wearing Apple Watch during various operating modes [19]. Tzeis et al. demonstrated that 171 adults wearing the Apple Watch under sensitive settings did not experience oversensing in EGM or CIED reprogramming [20]. The present study further demonstrated for the first time that there are no safety concerns of using KardiaMobile 6L and Apple Watch in pediatric patients with CIEDs.

### 4.3 Clinical Benefits and Limitations

Potential clinical benefits have been found with the use of portable smartphone‐based heart monitors for cardiac rhythm monitoring and detection of CIED dysfunction in adults. There were reports of the ability of a smart watch‐recorded ECG to detect intermittent loss of capture in an adult with a dual‐chamber pacemaker [21], pacemaker‐mediated tachycardia that was not recorded in the event logs of pacemaker [22], and malfunctioning of pacemaker lead despite apparent absence of abnormalities from chest X‐ray, echocardiogram, or pacemaker interrogation [23]. Our study provides further evidence that KardiaMobile 6L and Apple Watch can generate ECGs that enable physicians to identify non‐capture events, in particular ventricular non‐capture events, accurately in pediatric patients with CIEDs. This is of particular relevance given the potential challenges of communicating symptoms in children with CIEDs. Notwithstanding, the present study also highlights the potential challenges of smartphone‐based heart monitors for detecting non‐capture events. These devices utilize dry electrodes, as opposed to conventional ECG machines that rely on gel electrodes, rendering them more susceptible to motion artefacts and potential disruptions from sweat or bodily fluids during ECG recordings [24]. Moreover, the analysis of ECGs captured in digital format may be hindered by formatting constraints on smartphones or computers, potentially leading to the oversight of clinically significant long pauses. Physicians should be aware of these pitfalls when assessing non‐captures in ECGs recorded from these devices.

### 4.4 Integration With RM

The use of RM for patients with CIEDs has been advocated [25]. RM facilitates web‐based transmission of alerts to clinics, enhancing early detection of device dysfunction and reducing the need for frequent clinic visits. Lindsey et al. showed that while RM improved adverse event detection in pediatric patients, only 41% of triggered alerts corresponded to real actionable issues [26]. Additionally, Heidbüchel et al. found that early increases in pacing thresholds often went undetected by RM [27]. Cases of pacing non‐capture unnoticed by RM but identified during hospital visits have also been reported [28]. The findings of our study may suggest potential values of integrating portable smartphone‐based heart monitors into the RM program, especially in patients with cardiac devices that do not support RM, to improve the detection of adverse events.

### 4.5 Study Limitations

Our study has several limitations. First, this is a single‐center study. While this still represented the largest territory‐wide cohort of patients, subject recruitment was restricted to the relatively small number based on clinic follow‐up schedules amid the era of RM. Second, while the study included various types of CIEDs, a significant proportion of the subjects had epicardial leads manufactured by a single company. As a result, our findings might not fully represent the performance and safety of CIED models and configurations from different manufacturers. Third, because only a single device was programmed for unipolar sensing and pacing, this specific configuration is underrepresented in our cohort. Furthermore, pacemaker‐dependent patients and variations across different CIED types were not explored. Finally, while our study design focused specifically on the safety of portable smartphone‐based heart monitors, the presence of larger magnets in certain smartphones precludes any definitive conclusions regarding the safe use with paired smartphones or alternative external monitors. Addressing these limitations in future studies can provide a more comprehensive understanding of the clinical use of portable smartphone‐based heart monitors in patients with CIEDs.

## 5 Conclusions

In conclusion, KardiaMobile 6L and Apple Watch did not produce EMI that affected children and adolescents with CIEDs. The generated ECGs have enabled reliable measurement of heart rate and accurate identification of ventricular non‐captures in these patients.

## Author Contributions

Conception of study: Chun‐Lok Ho, Sit‐Yee Kwok, and Yiu‐Fai Cheung. Acquisition of data: Chun‐Lok Ho. Analysis or interpretation of data: Chun‐Lok Ho, Sit‐Yee Kwok, and Sabrina S. L. Tsao. Drafting of the article: Chun‐Lok Ho, Sit‐Yee Kwok, and Sabrina S. L. Tsao. Critical revision for important intellectual content: Sit‐Yee Kwok, Sabrina S. L. Tsao, and Yiu‐Fai Cheung. All authors had full access to the data, contributed to the study, approved the final version for publication, and take responsibility for its accuracy and integrity.

## Funding

The authors have nothing to report.

## Ethics Statement

The study was approved by the Central Institutional Review Board of the Hospital Authority, Hong Kong.

## Conflicts of Interest

The authors declare no conflicts of interest.

## Supporting information


**Figure S1:** Detection of ventricular non‐capture with Apple Watch.

**Table S1:** Summary of CIEDs, leads characteristics, and their programmed sensitivities.

## Data Availability

The data that support the findings of this study are available on request from the corresponding author. The data are not publicly available due to privacy or ethical restrictions.
