# Supplementary material for: Safety and Utility of Smartphone‐Based Heart Monitors in Pediatric Patients With Cardiovascular Implantable Electronic Devices
Source: J Cardiovasc Electrophysiol. 2026 Jul 19;37(11):2518–28. doi: 10.1111/jce.70444 (PMC13768786; doi:10.1111/jce.70444)

Supplementary figure 1

Detection of ventricular non-capture with Apple WatchTM.

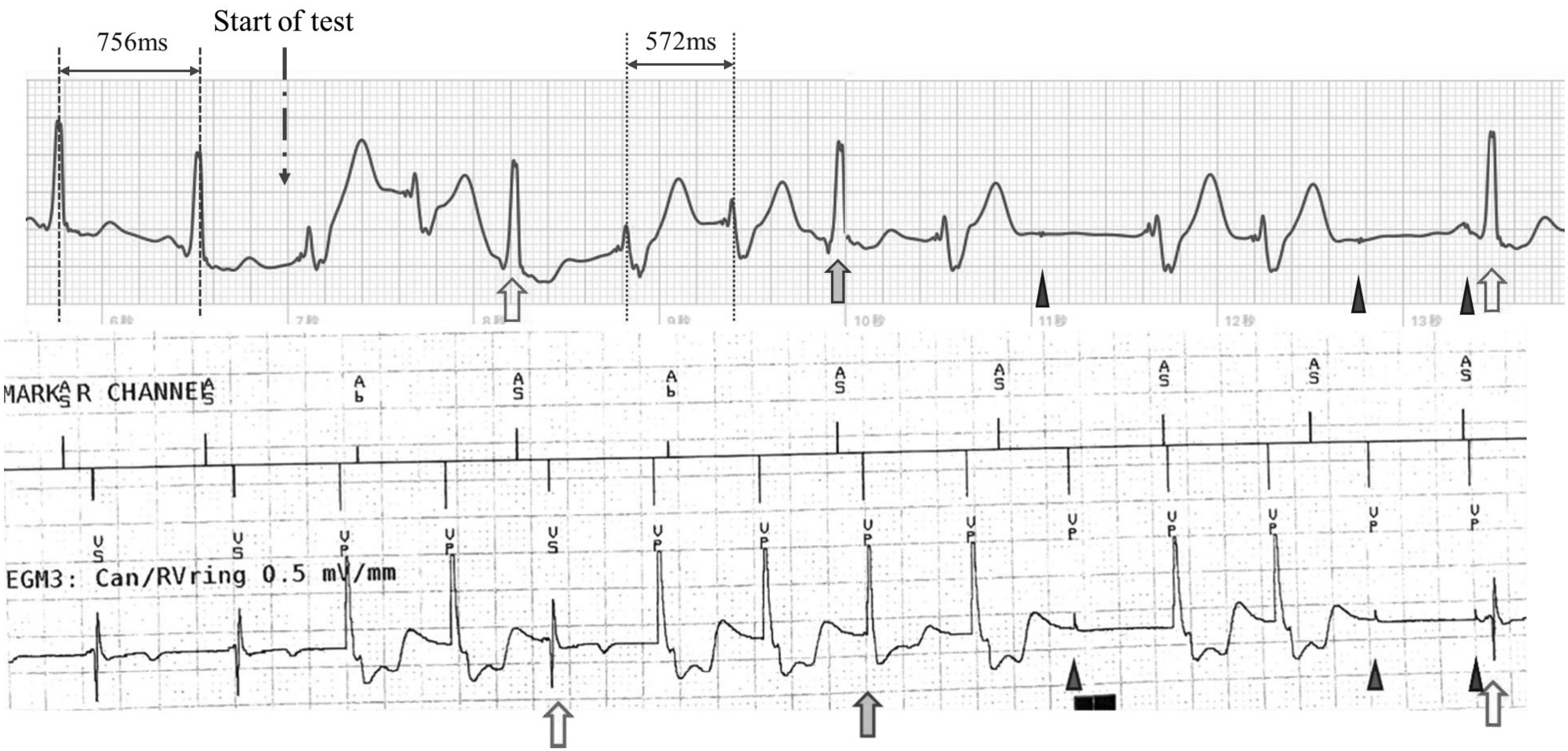

Supplement: Supplementary file 1 [file JCE-37-2518-s001.pdf]
